# Systematic Review and Case Report of a Left Gonadal Vein Anastomosing Hemangioma

**DOI:** 10.3390/jcm14093108

**Published:** 2025-04-30

**Authors:** Ilda Hoxhaj, Marco Piccino, Ugo Grossi, Valeria Maffeis, Alessandro Beleù, Francesca Baciorri, Giovanni Morana, Paolo Zanatta, Giacomo Zanus

**Affiliations:** 1 Surgery Unit 2, Department of Surgery, Regional Hospital Treviso AULSS 2 Marca Trevigiana, 31100 Treviso, Italy; ilda.hoxha31@gmail.com (I.H.); marco.piccino@aulss2.veneto.it (M.P.); giacomo.zanus@aulss2.veneto.it (G.Z.); 2 Department of Medical and Surgical Sciences, Fondazione Policlinico Universitario Agostino Gemelli, Istituto di Ricovero e Cura a Carattere Scientifico (IRCCS), 00168 Rome, Italy; 3 Department of Pathology, “Ca’ Foncello” Regional Hospital, 31100 Treviso, Italy; valeria.maffeis@aulss2.veneto.it (V.M.); francesca.baciorri@aulss2.veneto.it (F.B.); 4 Department of Radiology, Regional Hospital Treviso AULSS 2 Marca Trevigiana, 31100 Treviso, Italy; alessandro.beleu@aulss2.veneto.it (A.B.); giovanni.morana@aulss2.veneto.it (G.M.); 5 Department of Anesthesiology and Critical Care, Regional Hospital Treviso AULSS 2 Marca Trevigiana, 31100 Treviso, Italy; paolo.zanatta1@aulss2.veneto.it; 6 Department of Surgery, Oncology and Gastroenterology—DiSCOG, University of Padova, 35121 Padua, Italy

**Keywords:** anastomosing hemangioma, angiosarcoma, systematic review, mimicker

## Abstract

**Background/Objectives**: Anastomosing hemangioma (AH) is a rare, benign vascular tumor predominantly found in the genitourinary tract and often associated with impaired renal function. Due to its nonspecific radiological features, AH is frequently misinterpreted as a malignant vascular neoplasm, particularly angiosarcoma (AS), leading to potentially unnecessary surgical interventions. This study presents a systematic review of AH cases and describes a rare instance of retroperitoneal AH arising from the left gonadal vein, which was resected due to diagnostic uncertainty. **Methods:** A 68-year-old man underwent imaging for benign prostatic hyperplasia, incidentally revealing a 15-mm hypervascular retroperitoneal nodule adjacent to the left psoas muscle. Imaging findings, including moderate metabolic uptake on 18FDG-PET/CT, raised suspicion for AS. Given the diagnostic uncertainty and high-risk location, the multidisciplinary team (MDT) recommended surgical resection. Laparoscopic excision was performed, and histopathological analysis confirmed AH. The patient remained asymptomatic at a 22 month follow-up. In addition, a systematic review of 159 cases from 64 studies (2009–2024) was conducted to analyze radiological features, treatment approaches, and outcomes. **Results:** Among the reviewed cases, 68% were incidentally diagnosed, with AH occurring predominantly in the genitourinary system (70%), especially in the kidney, adrenal gland, and ovary. Chronic kidney disease (CKD) was present in 23.3% of cases, while 19.5% had a history of malignancy. Imaging was inconclusive in differentiating AH from malignancies: CT (71.9%) and MRI (6.1%) were the most used modalities, but none could reliably exclude AS. Management strategies included upfront surgical resection in 85%, while a growing proportion (9%) of cases underwent biopsy-based observation rather than immediate surgery. No cases were followed with imaging alone. **Conclusions:** AH remains a diagnostic challenge due to its overlap with malignant vascular tumors. While surgical excision is often performed, our review highlights an increasing trend toward conservative management with biopsy-based diagnosis. Improved awareness and the integration of histopathology, molecular markers, and MDT-based decision-making are crucial to prevent overtreatment in cases of suspected AH.

## 1. Introduction

Vascular lesions cover a wide range of conditions, from noncancerous arteriovascular malformations to aggressive, malignant angiosarcomas; hemangiomas, a benign form of vascular tumor, first described by Virchow in 1867, are the most common type of vascular neoplasm and can occur in any organ or tissue [1]. The development of hemangiomas can be influenced by factors such as age, gender, and the tumor’s location, and the specific subtype of hemangioma—such as capillary, venous, or epithelioid—also play a role. Multifocal hemangiomas are present in cases of systemic angiomatosis, as seen in Sturge–Weber and Klippel–Trénaunay syndromes [1]. The most recent entity, the anastomosing hemangioma (AH), as a subtype of capillary hemangioma, has been recognized as a distinct vascular tumor with unique histological features. First reported by Montgomery and Epstein in 2009, AH was initially described in the genitourinary tract, particularly in the kidney and retroperitoneum [1]. AH predominantly affects visceral organs and deep soft tissues, with a marked preference for the genitourinary system, particularly the kidneys, adrenal glands, and ovaries [2]. It is often discovered incidentally during imaging studies performed for unrelated conditions. Clinically, AH presents as a well-circumscribed, brown-colored nodule, and is usually asymptomatic, although some patients may report mild pain or hematuria [3].

Histologically, AH consists of an anastomosing network of capillary-sized vascular channels, resembling splenic sinusoids. The endothelial cells lining these vessels show minimal atypia, but the growth pattern may mimic well-differentiated angiosarcoma (AS), leading to potential misdiagnosis [4,5]. Imaging techniques such as contrast-enhanced computed tomography (CT) and magnetic resonance imaging (MRI) often fail to differentiate AH from malignant vascular tumors [6].

Since its common location in the renal district, AH could be misidentified as a malignant renal tumor, especially as renal cell carcinoma (RCC), in imaging studies, often resulting in futile nephrectomies. Moreover, at a histopathological level, intrarenal hemangiomas can also appear similar to low-grade clear cell renal cell carcinoma, hemangioblastoma, and even RCC. This diagnostic uncertainty often leads to unnecessary surgical resections. While fine-needle aspiration (FNA) biopsy can aid in diagnosis, its accuracy in distinguishing AH from AS or RCC remains limited, and potential risks such as bleeding or damage to surrounding structures must also be considered [7].

Given these diagnostic challenges, a comprehensive understanding of AH’s clinical, radiological, and histological characteristics is essential to optimize patient management. In this study, we present a systematic review of the published cases of AH, evaluating its diagnostic approach and treatment strategies. Additionally, we report a rare case of AH arising along the left gonadal vein, highlighting its clinical and pathological features.

## 2. Materials and Methods

We conducted a systematic review of published case reports on the management of AH, following the Preferred Reporting Items for Systematic Reviews and Meta-Analyses (PRISMA) guidelines [8]. This review was not registered in any systematic review database or registry.

### 2.1. Eligibility Criteria

To define the scope of our review, we applied the PICOS framework without time restrictions:

- Population: Patients diagnosed with anastomosing hemangioma.
- Intervention/Exposure: Treatment approach, including surgical resection or conservative management.
- Comparison: Not applicable.
- Outcome: Histopathological diagnosis and follow-up.
- Study Design: Case reports or case series.

We included peer-reviewed case reports and case series that provided primary data on AH, regardless of the publication date. We excluded editorials, comments, conference papers, perspectives, short communications, and narrative reviews. Additionally, we excluded the reports of AH located in the head, neck, limbs, or skin, as their management differs from thoraco-abdominal cases.

### 2.2. Search Strategy

Two independent researchers (IH, MP) systematically searched PubMed, ISI Web of Science, and Scopus for articles published from inception until 1 September 2024.

A structured search strategy was developed using Medical Subject Headings (MeSH) terms and free-text keywords, combined with Boolean operators. The PubMed search string included: “hemangioma” [MeSH], “anastomosing hemangioma”, and “vascular hemangioma”.

This search string was adapted for ISI Web of Science and Scopus. The search was restricted to studies published in English, Italian, Spanish, or French, with no further limitations.

To ensure completeness, the reference lists of included articles were manually screened for additional relevant studies.

All retrieved articles were imported into Mendeley for reference management, and duplicates were removed. The remaining articles were uploaded to Rayyan (Qatar Computing Research Institute) for screening. Two independent researchers (IH, MP) screened the titles and abstracts, followed by full-text review. Discrepancies were resolved through discussion, with the involvement of a third researcher (UG) when necessary.

### 2.3. Data Extraction and Synthesis

To ensure consistency in data collection, we developed a standardized data extraction form tailored to the objectives of this systematic review. This form captured key information from each included study, such as the first author, title, and publication year, to accurately catalog each article. Additionally, we recorded the study characteristics, including the country of origin and study design. For patient-related information, we documented the population characteristics, such as sample size, sex, and age, to understand the demographic distribution of AH cases. Particular attention was given to diagnostic methods, including imaging techniques (CT, MRI, PET scans) and biopsy results, to assess the strategies used for AH identification. We also categorized treatment approaches, distinguishing between surgical intervention (e.g., total or partial organ resection) and conservative management (e.g., observation after biopsy). Finally, we extracted follow-up data, including duration and reported outcomes, to evaluate disease progression and recurrence rates.

All data were systematically compiled into Excel spreadsheets.

We used SPSS (IBM SPSS Statistics Version 21, International Business Machines Corporation, Armonk, NY, USA) for data analysis. Categorical variables were presented as number (%) and continuous variables as mean (standard deviations [SD]) when normally distributed or median (interquartile range [IQR]) if not. We used χ^2^ and Fisher’s exact tests to compare categorical variables and the *t* test or the Mann-Whitney U test to compare continuous variables. Significance was set at *p* < 0.05.

### 2.4. Quality Assessment

To assess the methodological rigor of the included studies, we applied the Joanna Briggs Institute (JBI) Critical Appraisal Checklist [9], a widely used tool designed for evaluating case reports and case series. This checklist provides a structured framework to determine the reliability of individual studies by examining key aspects such as the clarity of case descriptions, the completeness of patient history reporting, the appropriateness of diagnostic and therapeutic approaches, and the assessment of clinical outcomes.

Studies that satisfied at least 75% of the criteria were considered to be of high methodological quality, demonstrating strong reporting standards and reliable data. Those meeting between 50% and 74% of the criteria were categorized as moderate quality, indicating a reasonable level of rigor but with some methodological limitations. In contrast, studies that fulfilled fewer than 50% of the criteria were classified as low quality, suggesting a higher risk of bias and insufficient methodological robustness.

## 3. Results

### 3.1. Systematic Review Results

**Figure 1:**
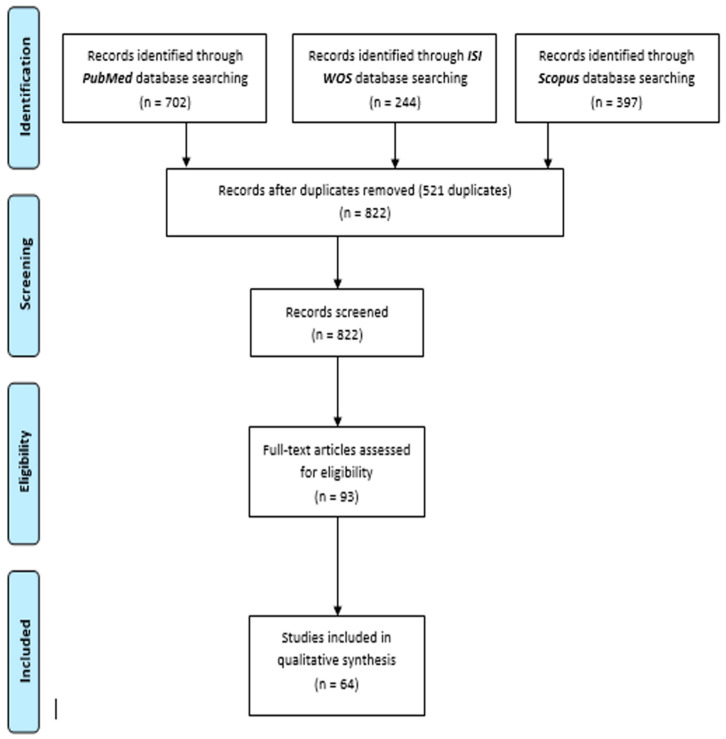
PRISMA diagram.

The initial database search retrieved 822 articles, which were independently screened by two researchers (IH and MP) based on their titles and abstracts. After this preliminary assessment, 93 articles were selected for full-text review. Following a thorough evaluation, 64 studies published between 2009 and March 2024 met the inclusion criteria and were incorporated into the systematic review (Figure 1). No additional studies were identified through manual reference list screening.

Collectively, these studies reported data on 159 adult patients diagnosed with thoracic or abdominal anastomosing hemangiomas (AH) (Table 1). The methodological quality of the included studies varied, with most providing clear documentation of the patient demographics, clinical presentation, and key takeaways. Nearly 80% of the studies offered detailed descriptions of the diagnostic approaches, treatment modalities, and clinical outcomes, ensuring a comprehensive assessment of AH management. Based on the quality appraisal, the majority of the included articles were classified as having good to moderate methodological quality, reinforcing the reliability of the available evidence.

The majority of the included studies were conducted in the United States (57.9%), followed by China (15.7%), and various European countries (13.2%). A smaller proportion originated from Oceania (4.4%), while isolated cases were reported from Canada, Japan, India, Brazil, Saudi Arabia, Singapore, and South Korea.

Among the 159 patients included, a slight majority were female (51%), with a median age of 58 years (IQR: 48–67), ranging from 15 to 85 years old. A history of renal impairment was reported in 23.3% of cases, while 6.3% had a diagnosis of arterial hypertension. Additionally, 19.5% of patients had a previous malignancy, highlighting a potential association between AH and oncological history.

**Figure 2:**
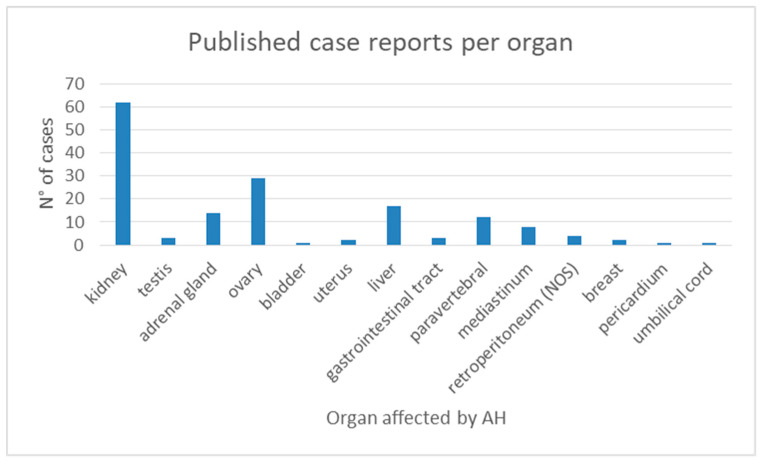
Distribution of AH by site of origin.

The genitourinary tract was the most commonly affected site, accounting for 70% of cases. Specifically, AH was most frequently found in the kidneys (62 cases), followed by the adrenal glands (14 cases), ovaries (29 cases), testes (3 cases), uterus (2 cases), and bladder (1 case) (Figure 2). Outside the genitourinary system, the most frequently involved organ was the liver (17 cases), followed by the paravertebral space (12 cases), mediastinum (8 cases), retroperitoneum (4 cases), gastrointestinal tract (3 cases), breast (2 cases), pericardium (1 case), and umbilical cord (1 case).

The mean diameter of the anastomosing hemangioma (AH) lesions was 26.5 mm, with a size range spanning from 1 mm to 110 mm. In most cases (68%), AH was incidentally discovered, often during imaging for unrelated conditions. When symptomatic, patients typically reported regional pain and mass effect, with hematuria and lower urinary tract discomfort being less common.

The interval from diagnosis to final treatment varied widely, with a mean duration of 4.8 months (SD: 17.1), ranging from 1 to 120 months. Regarding diagnostic imaging, CT was the most frequently employed modality (71.9%), followed by MRI (6.1%). A combination of CT and MRI was used in 16.7% of cases, while only four patients underwent FDG-PET-CT, including the present case, and a single case was assessed with Ga-PET-CT.

**Figure 3:**
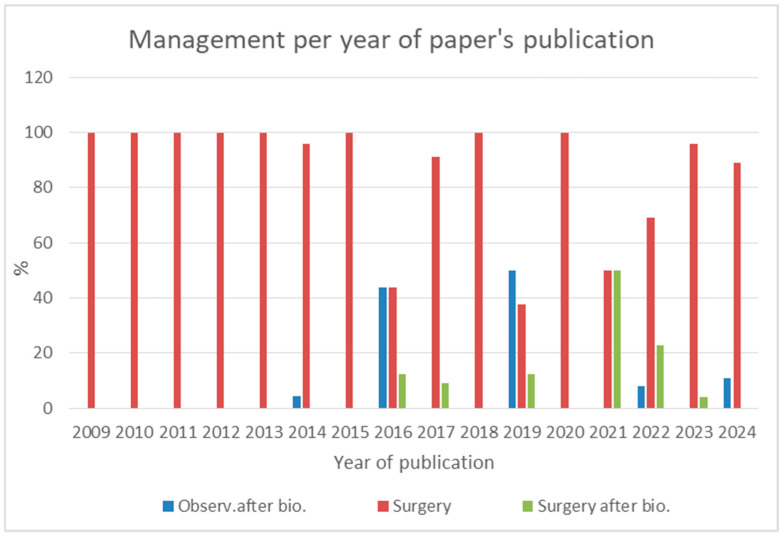
Management of anastomotic hemangioma over time.

**Figure 4:**
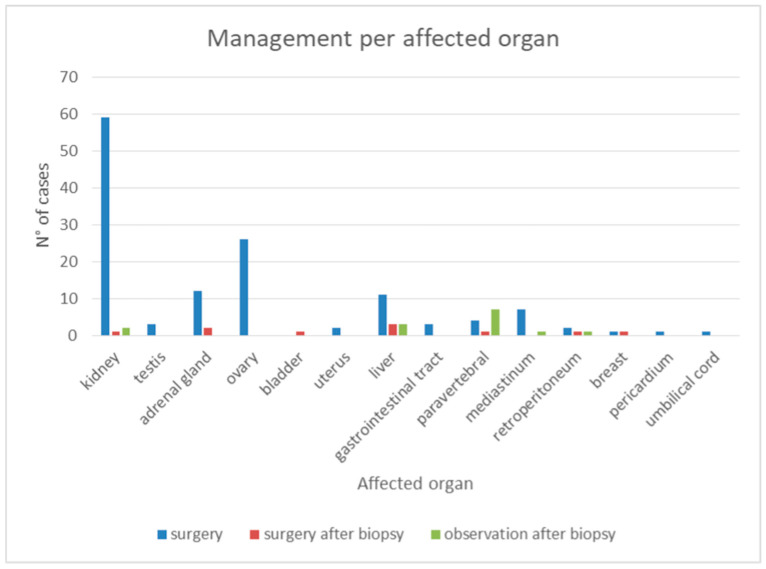
Management of anastomotic hemangioma according to site of origin.

In terms of the treatment strategies, the vast majority of patients (85%) underwent upfront surgical resection. A smaller proportion (6%) had surgery following an initial diagnostic biopsy, while 9% were managed conservatively with periodic observation. Notably, no cases were followed solely with imaging-based surveillance. Given the high rate of upfront surgical resections, it is reasonable to infer that, in most cases, surgery was performed under a clinical suspicion of malignancy. To provide insight into treatment trends over time, we analyzed the evolution of management strategies across different years (Figure 3 and Figure 4).

When surgery was performed, the extent of resection varied depending on the lesion’s characteristics and location. In 21% of cases, only the lesion itself was excised, whereas in 13%, a partial resection of the affected organ was necessary. The majority of cases (60%) required total resection of the involved organ, while a smaller subset (6%) underwent an extended local resection.

Postoperative morbidity was minimal, with complications occurring in only 1% of cases, which were all classified as Grade 2 according to the Clavien–Dindo classification. No cases of recurrence or disease progression were reported during the follow-up, reinforcing the benign nature of AH. The median follow-up period was 14 months (IQR = 8.0–32.8 months).

AH was incidentally detected in 76% of patients with a history of malignancy and in 65% of those without an oncological background. Similarly, AH was identified in 65.3% of patients without renal disease and in 72.7% of those with renal impairment, with mild, moderate, or severe stages of renal dysfunction. Specific diagnostic criteria for renal impairment, such as creatinine elevation or formal chronic kidney disease (CKD) staging, were not consistently reported in the included studies.

However, renal impairment was significantly associated with the AH location. Patients with genitourinary AH had a higher prevalence of renal disease (32%) compared to those with non-genitourinary AH (2.2%) (*p* < 0.001). This association was even stronger when comparing renal AH to all other locations, with 54.8% of renal AH cases occurring in patients with renal impairment, compared to only 8.1% in other sites (*p* < 0.001).

The association between AH and prior malignancies also varied by anatomical site. The prevalence of a history of malignancy was highest in AH of the ovary (34.5%), followed by the liver (29.4%), adrenal gland (21.4%), and kidney (19.4%). Notably, no previous malignancies were reported in AH arising in other locations.

### 3.2. Case Presentation

We report a case of anastomosing hemangioma (AH) arising along the left gonadal vessels, documented following the SCARE guidelines [68].

A 68-year-old man, with a body mass index of 29.2 kg/m^2^, was referred to our tertiary medical center for further evaluation of a retroperitoneal mass that was incidentally detected during a routine follow-up for benign prostatic hyperplasia. The patient was in good general health and reported no symptoms related to the mass.

His medical history was notable for hypertension, which was well controlled with medication, and a laparoscopic appendectomy performed three years prior. He had no known history of renal dysfunction, and both his personal and family history were negative for oncological diseases.

As part of his benign prostatic hyperplasia follow-up, the patient underwent a multiparametric prostate MRI due to an elevated prostate-specific antigen level. The scan was performed using a Magnetom Avanto Fit 1.5T scanner (Siemens Healthineers, Erlangen, Germany). The protocol primarily focused on the prostate and adjacent structures, using a restricted field of view (FOV) for detailed imaging. However, panoramic scans with a wider FOV were also included in the pelvic region, which led to the incidental identification of a suspicious 15-mm nodule located anterior to the left psoas muscle.

**Figure 5:**
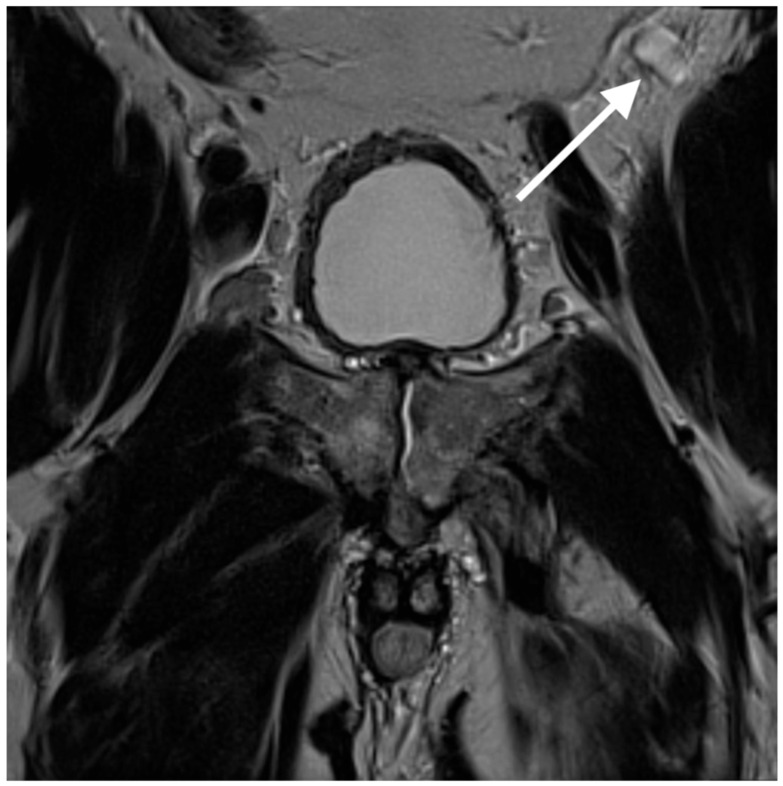
Coronal T2 HASTE sequence demonstrating a uniformly hyperintense nodule anterior to the left iliopsoas muscle (white arrow).

In the pre-contrast phase, the coronal T2-weighted HASTE sequence demonstrated a homogeneously hyperintense lesion (Figure 5, white arrow), a characteristic feature of many hemangiomas.

The axial panoramic T1-weighted DIXON out-of-phase sequences showed no signal drop, ruling out significant fat content within the lesion.

**Figure 6:**
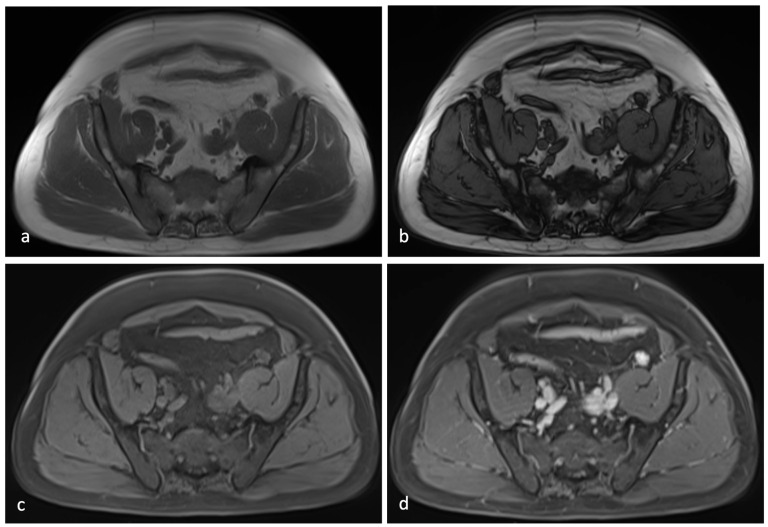
Axial DIXON T1-weighted sequences in (**a**) and out-of-phase (**b**). Pre-contrast T1w VIBE sequences (**c**) and post-contrast graphic sequences in late phase (**d**).

In the late post-contrast phase, after completing the prostate-specific scans, the axial panoramic T1-weighted VIBE sequence—performed with a wide field of view (FOV)—revealed vivid and homogeneous enhancement of the nodule (Figure 6).

Other imaging sequences, including dynamic post-contrast T1-weighted VIBE and diffusion-weighted imaging (DWI), did not fully encompass the lesion within the scanned field.

Given the suspicion of lymphadenopathy, a contrast-enhanced CT scan of the abdomen was performed. The scan was acquired using a Somatom Flash (Siemens Healthineers, Erlangen, Germany), with thin-slice (1.25 mm) acquisitions at 120 kV.

**Figure 7:**
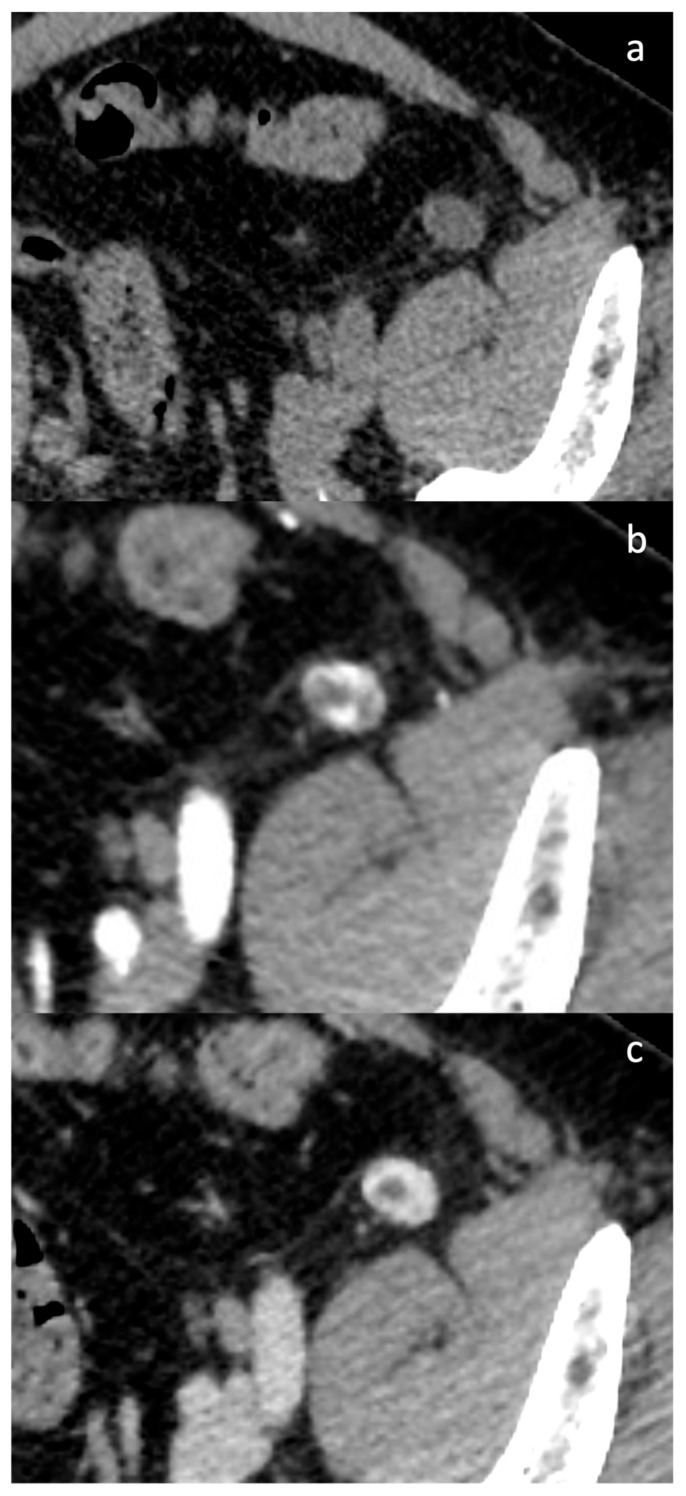
Axial CT scan details in the pre-contrast phase (**a**), arterial phase (**b**), and venous phase (**c**) with a demonstration of peripheral centripetal globular enhancement.

In the pre-contrast phase, the 15 mm nodule was clearly identified, demonstrating a solid, homogeneous density of 25 ± 12 Hounsfield Units (HU), without calcifications. Following contrast administration (Ultravist 370, Bayer, Leverkusen, Germany), the arterial phase revealed a vivid enhancement with a predominantly peripheral, globular centripetal pattern, reaching an average intensity of 116 ± 61 HU. In the venous phase, further contrast filling was observed, reaching 125 ± 50 HU, with a persistent small hypovascular central area (Figure 7). No additional pathological findings were detected.

To further investigate potential malignancy, a colonoscopy was performed, which identified the following: two polyps in the distal sigmoid colon, both diagnosed as hyperplastic polyps, and two polyps in the ascending colon, both confirmed as tubular adenomas with low-grade dysplasia (<10 mm).

Additionally, serum tumor markers—CEA, CA 15.3, CA 125, CA 19-9, and AFP—were all within normal limits, providing no immediate oncological concern.

An 18FDG-PET/CT was performed to assess metabolic activity. The scan demonstrated moderate uptake (SUV max 4.13) at the 15 mm nodular formation, raising concern for a potential malignancy.

A multidisciplinary team (MDT) meeting was convened to evaluate the findings. While the imaging features were not definitively suggestive of malignancy, a neoplastic process could not be excluded. Given the mass’s retroperitoneal location, adjacency to critical structures, and its relatively small size, a CT-guided fine-needle aspiration (FNA) biopsy was considered high risk. Concerns included insufficient tissue sampling and potential peri-procedural complications. Consequently, surgical resection was deemed the most appropriate approach.

The patient subsequently underwent a laparoscopic resection of the suspected mass. Intraoperatively, the lesion was precisely located in the retroperitoneal space, adjacent to the left gonadal vein, cranially to the internal inguinal ring.

The excised nodule appeared fleshy, soft, and well-demarcated, yet lacked a capsule, a feature commonly associated with benign vascular lesions. It exhibited a mahogany brown coloration, a characteristic suggestive of a highly vascular nature.

A striking intraoperative observation was the immediate reduction in volume upon ligation of its blood supply, further reinforcing the suspicion of a vascular lesion.

Following resection, the specimen was sent for intraoperative frozen section analysis. The 1 cm well-demarcated nodule exhibited a hemorrhagic, spongy cut surface with no apparent signs of malignancy. The histopathological assessment of the frozen section confirmed a vascular lesion, in line with the initial macroscopic impression.

The patient’s postoperative recovery was uneventful, and he was discharged the following day without complications.

**Figure 8:**
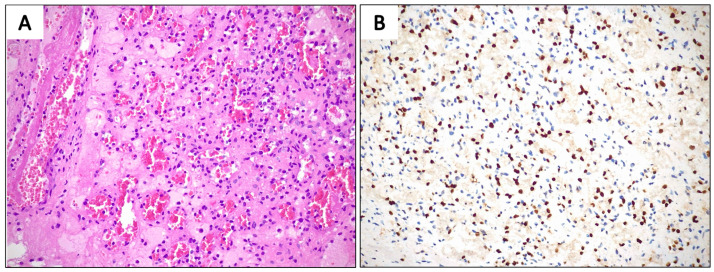
Hematoxylin and eosin staining of the lesion (**A**) and corresponding positive nuclear marker ERG (**B**).

The final histological examination of the resected specimen revealed a well-demarcated proliferation of small to medium-sized vessels, which were embedded within a framework of non-endothelial supporting cells (Figure 8A). This confirmed the diagnosis of an AH.

Microscopic examination revealed scattered hobnailed endothelial cells, which lacked cytological atypia or mitotic activity. Additionally, fibrin microthrombi were identified within the vascular spaces, a feature commonly observed in benign vascular proliferations.

Immunohistochemical staining confirmed the endothelial origin of the lesion, with ERG positivity (Figure 8B). In contrast, pancytokeratin, D2-40, and HHV-8 were negative, effectively ruling out mesothelial tumors and Kaposi sarcoma. The overall morphology and immunophenotypic profile strongly supported the diagnosis of an AH.

The patient underwent close postoperative monitoring, with CT imaging and tumor marker testing performed at four months post-resection. These assessments showed no evidence of recurrence.

At 22 months post-surgery, the patient remained asymptomatic, with no signs of disease recurrence on follow-up imaging. The absence of relapse further reinforced the benign nature of AH and supports the appropriateness of the surgical approach in cases where malignancy cannot be confidently excluded preoperatively.

## 4. Discussion

### 4.1. Key Findings from the Systematic Review

This systematic review consolidates the current understanding of AH as a rare benign vascular neoplasm often mistaken for malignancy due to its histological and radiological features. A total of 159 cases from 64 published articles were analyzed, with the majority of cases incidentally detected in the genitourinary tract (70%), particularly the kidneys, adrenal glands, and ovaries. While AH has been reported in extra-genitourinary sites, including the liver, retroperitoneum, mediastinum, and the paravertebral space, the diagnostic and management challenges remain consistent across different anatomical locations.

### 4.2. Demographics and Risk Factors

AH predominantly affects middle-aged adults, with a median age of 58 years (IQR 48–67), though cases have been reported from 15 to 85 years. There is a slight female predominance (51%), likely influenced by the high prevalence of ovarian AH. Importantly, 19.5% of patients had a history of malignancy, raising questions about a possible association between AH and oncologic backgrounds. The highest rates of prior malignancies were observed in ovarian AH (34.5%), hepatic AH (29.4%), adrenal AH (21.4%), and renal AH (19.4%), suggesting that AH may preferentially develop in tumor-prone environments. However, the overall incidence of incidental AH detection was comparable between oncologic and non-oncologic patients (76% vs. 65%, *p* = 0.18), indicating that AH occurrence is not necessarily increased in cancer patients [50] but rather that oncologic follow-up imaging may contribute to higher detection rates.

A particularly strong association was observed between AH and renal impairment [50,69,70]. In our review, 23.3% of cases had some degree of renal dysfunction, and among patients with renal AH, 54.8% had CKD. This was significantly higher than the estimated 14% CKD prevalence in the general adult population and suggests that impaired renal function may be a predisposing factor for AH development, particularly in the kidneys. However, the rate of incidental AH detection remained similar between patients with and without CKD (72.7% vs. 65.3%, *p* = 0.29), indicating that increased imaging in CKD patients does not necessarily lead to a higher diagnosis rate of AH.

### 4.3. Diagnostic Challenges and Imaging Limitations

One of the major challenges in AH management is its nonspecific imaging features, which can closely mimic malignant tumors, particularly AS. In our review, most AH lesions were incidentally found (68%), with regional pain or mass effect being the most common symptoms when present. The mean lesion size was 26.5 mm, ranging from 1 mm to 110 mm, with no clear correlation between the size and malignancy suspicion.

Imaging studies remain inconclusive in differentiating AH from malignant vascular tumors [51,69]. CT scans were the most frequently used modality (71.9%), showing well-circumscribed, hyperdense nodules, often with persistent enhancement in venous and delayed phases. MRI was employed in 6.1% of cases, typically revealing hypointensity on T1-weighted imaging (T1WI) and hyperintensity on T2-weighted imaging (T2WI), resembling other benign hemangiomas.

FDG-PET/CT was performed in only four cases (including our case), highlighting the low utility of PET in distinguishing AH from malignancy, as AH may present with both hypermetabolic (“hot”) and hypometabolic (“cold”) patterns [71].

Interestingly, our case report is the first documented instance where an AH lesion was evaluated with preoperative FDG-PET/CT, showing a moderate uptake (SUV max 4.13), which raised suspicion for malignancy. This underscores the lack of specificity of PET imaging in AH cases, further reinforcing the diagnostic uncertainty surrounding these lesions.

### 4.4. Histopathological Considerations and Differential Diagnosis

Histologically, AH is composed of anastomosing capillary-sized vascular channels resembling splenic sinusoids, lined by hobnailed endothelial cells [50]. In our systematic review, all cases demonstrated benign histological features, with no reported instances of atypia, necrosis, or significant mitotic activity. However, differentiating AH from well-differentiated AS remains a critical challenge [69].

Immunohistochemically, AH exhibits CD31, CD34, and ERG positivity, but it is negative for HHV-8, CD8, and D2-40, which helps exclude Kaposi sarcoma and lymphatic-derived lesions. A low Ki-67 proliferation index (<5%) further supports the benign nature of AH [36].

Recent molecular studies have suggested that AH harbors activating mutations in the GNAQ gene, similar to other benign vascular tumors but absent in AS. While not yet widely implemented, molecular profiling may serve as a future diagnostic tool to differentiate AH from malignant vascular tumors, particularly in small biopsy samples [72,73].

### 4.5. Evolution of AH Management: Shift Towards Conservative Approaches

Historically, AH was primarily managed with upfront surgery, given the uncertainty surrounding its benign nature. Our review confirms that 85% of cases underwent immediate surgical resection, with 6% having surgery following an initial biopsy, and only 9% managed conservatively with observation.

However, a notable shift in management trends has emerged over the past decade. Indeed, before 2015, nearly all AH cases were surgically resected. Since 2016, conservative management has been increasingly considered, particularly in non-genitourinary AH cases where surgical risk is higher. By 2022, the rate of resection had declined significantly (to as low as 37.5%), reflecting increased awareness and improved diagnostic strategies.

The primary concern regarding biopsy-based diagnosis is sampling error, as well-differentiated AS can mimic AH in focal areas, and some AHs may demonstrate subtle infiltrative features, leading to misinterpretation as malignancy. While biopsy plays an important role in selected cases, a combination of imaging, histology, and molecular markers is likely the most effective strategy for AH diagnosis.

### 4.6. Lessons from Our Case Report

Our case exemplifies the diagnostic dilemma posed by AH, particularly when incidentally detected in an atypical location (along the left gonadal vein). The decision-making process in our case was influenced by: (a) non-specific imaging findings, including moderate FDG-PET uptake, which raised suspicion for malignancy; (b) concerns regarding biopsy accuracy, given the small size (15 mm) and deep retroperitoneal location; and (c) MDT consensus, favoring surgical resection over high-risk biopsy.

The absence of recurrence at a 22 month follow-up further reinforces the benign nature of AH and supports the appropriateness of surgical resection when malignancy cannot be confidently excluded preoperatively.

### 4.7. Lessons from the Systematic Review

Anastomotic hemangioma should be considered for differential diagnosis in the following clinical scenarios:

- Middle-aged adult patient, no difference in sex;
- A solid homogeneous well circumscribed lesion, without calcifications, with a characteristic CT and MRI contrast enhancement, as described in many case reports in the literature;
- Minimal modifications in size during time;
- Incidental discovery in asymptomatic patient;
- Location in the genitourinary tract mainly, but also the liver and paravertebral space;
- A history of renal impairment or ESRD;
- Previous or coexistent oncological disease.

After an AH diagnosis is made, the subsequent course will be benign; during follow-up, remember the potential association between AH and oncological disease, in particular if the AH is located in the ovary, liver, adrenal gland, or kidney.

### 4.8. Clinical Implications and Future Directions

This systematic review highlights several key implications for AH diagnosis and management. Imaging remains unreliable in distinguishing AH from malignant vascular tumors, emphasizing the need for improved non-invasive diagnostic tools. AH is frequently associated with renal impairment, suggesting a possible link between CKD and AH development. The high prevalence of AH in oncologic patients raises the question of whether AH represents a secondary phenomenon in tumor-prone environments. Molecular profiling, including GNAQ mutation analysis, may enhance diagnostic accuracy and prevent unnecessary surgeries.

As our understanding of AH evolves, a risk-adapted approach integrating imaging, histology, molecular markers, and multidisciplinary decision-making will be crucial in optimizing patient management while minimizing overtreatment.

## 5. Conclusions

Given the growing recognition of AH in both oncologic and non-oncologic populations, a risk-adapted approach—incorporating clinical presentation, imaging, biopsy findings, and molecular insights—is essential for optimal patient management. Future research should further clarify the pathophysiological links between AH, renal impairment, and oncologic backgrounds, enhancing diagnostic precision and treatment decision-making.

## Figures and Tables

**Table 1:** Characteristics of included studies.

Source	Area	Incidental	Organ	Age, Years	Sex	Malignancy in Medical History	Renal Imp.	HTN	Diagnosis to Treatment Months	Imaging Performed	Size Max, mm	Treatment ^¥^	Surgery *	Postoperative Morbility	Follow Up, Months
Montgomery E et al., 2009 [1]	US	ns	kidney	74	m	n	n	n	1	ns	15	2	c	0	36
Montgomery E et al., 2009 [1]	US	ns	kidney	75	f	n	n	n	1	ns	20	2	c	0	ns
Montgomery E et al., 2009 [1]	US	ns	kidney	65	f	n	n	n	1	ns	20	2	a	0	8
Montgomery E et al., 2009 [1]	US	ns	kidney	49	m	n	n	n	1	ns	13	2	c	0	12
Montgomery E et al., 2009 [1]	US	ns	testis	54	m	n	n	n	1	ns	15	2	c	0	8
Montgomery E et al., 2009 [1]	US	ns	testis	49	m	n	n	n	1	ns	17	2	c	0	12
Kryvenko O et al., 2011 [10]	US	y	ovary	70	f	y	n	n	1	CT	2	2	c	0	25
Kryvenko O et al., 2011 [10]	US	y	ovary	49	f	n	n	n	1	CT	1	2	c	0	16
Kryvenko O et al., 2011 [10]	US	y	ovary	77	f	n	n	n	1	CT	11	2	c	0	32
Kryvenko O et al., 2011 [10]	US	y	kidney	51	f	n	y	n	1	CT	10	2	c	0	122
Kryvenko O et al., 2011 [10]	US	y	kidney	39	m	y	y	n	1	CT	50	2	c	0	6
Kryvenko O et al., 2011 [10]	US	y	kidney	67	f	n	n	n	1	CT	12	2	c	0	6
Kryvenko O et al., 2011 [10]	US	y	kidney	54	f	n	y	n	1	CT	12	2	c	0	3
Tran AT et al., 2012 [11]	US	n	kidney	61	m	n	n	n	1	CT	21	2	c	0	ns
Ross M et al., 2012 [12]	US	y	adrenal g.	49	m	n	y	n	1	CT	37	2	d	0	ns
Metha V et al., 2012 [13]	US	ns	kidney	49	m	n	y	n	1	CT	20	2	c	0	3
Metha V et al., 2012 [13]	US	ns	kidney	55	m	n	y	n	1	CT	6	2	c	0	3
Metha V et al., 2012 [13]	US	ns	kidney	45	m	n	y	n	1	CT	19	2	c	0	12
Lin J et al., 2013 [14]	US	y	liver	64	f	n	n	n	1	ns	30	2	b	0	67
Lin J et al., 2013 [14]	US	y	liver	62	f	y	n	n	1	ns	24	2	b	0	14
Lin J et al., 2013 [14]	US	y	g.i. tract	70	f	n	n	n	1	ns	2	2	a	0	ns
Lin J et al., 2013 [14]	US	n	g.i. tract	68	m	n	n	n	1	ns	48	2	b	0	8
Lin J et al., 2013 [14]	US	y	liver	48	m	y	n	n	1	ns	20	2	b	0	18
Lin J et al., 2013 [14]	US	n	liver	71	f	n	n	n	1	ns	60	2	b	0	96
Zhao M et al., 2013 [2]	China	y	kidney	48	m	y	n	n	1	CT, MRI	23	2	b	0	12
Heidegger I et al., 2014 [15]	EU	n	kidney	56	m	n	n	n	1	CT	70	2	c	0	120
Heidegger I et al., 2014 [15]	EU	n	kidney	56	m	n	n	n	1	CT	70	2	c	0	144
Tao L et al., 2014 [16]	China	y	kidney	32	m	n	n	n	1	CT	34	2	c	0	21
Chou S et al., 2014 [17]	Oceania	y	kidney	50	f	y	y	n	1	CT	10	2	c	0	14
Chou S et al., 2014 [17]	Oceania	y	kidney	60	m	n	y	y	1	CT	28	2	c	0	8
Tahir M et al., 2014 [18]	Oceania	y	kidney	57	m	n	n	n	1	CT	46	2	c	0	12
Downes MR et al., 2014 [19]	Canada	y	kidney	59	f	y	n	n	1	CT	45	2	c	0	ns
Downes MR et al., 2014 [19]	Canada	y	kidney	28	m	y	n	n	36	CT	13	1		0	ns
Kryvenko ON et al., 2014 [20]	US	n	kidney	68	f	n	y	n	1	CT	15	2	c	0	ns
Kryvenko ON et al., 2014 [20]	US	y	kidney	51	f	n	y	n	1	CT	10	2	c	0	ns
Kryvenko ON et al., 2014 [20]	US	n	kidney	54	f	y	y	n	1	CT	11	2	c	0	ns
Kryvenko ON et al., 2014 [20]	US	y	kidney	29	m	n	y	n	1	CT	13	2	c	0	ns
Kryvenko ON et al., 2014 [20]	US	y	kidney	40	m	y	y	n	1	CT	3	2	c	0	ns
Kryvenko ON et al., 2014 [20]	US	n	kidney	34	m	y	y	n	1	CT	13	2	c	0	ns
Kryvenko ON et al., 2014 [20]	US	y	kidney	62	m	y	y	n	1	CT	7	2	c	0	ns
Kryvenko ON et al., 2014 [20]	US	y	kidney	40	m	n	y	n	1	CT	28	2	c	0	ns
Kryvenko ON et al., 2014 [20]	US	y	kidney	46	m	n	y	n	1	CT	16	2	c	0	ns
Kryvenko ON et al., 2014 [20]	US	y	kidney	60	m	y	y	n	1	CT	12	2	c	0	ns
Kryvenko ON et al., 2014 [20]	US	n	kidney	49	m	n	y	n	1	CT	35	2	c	0	ns
Kryvenko ON et al., 2014 [20]	US	ns	kidney	49	m	n	y	n	1	CT	13	2	c	0	ns
Kryvenko ON et al., 2014 [20]	US	n	kidney	66	m	y	y	n	1	CT	30	2	c	0	ns
Kryvenko ON et al., 2014 [20]	US	y	kidney	15	m	n	y	n	1	CT	7	2	c	0	ns
Kryvenko ON et al., 2014 [20]	US	n	kidney	17	m	y	y	n	1	CT	28	2	c	0	ns
Zhang W et al., 2015 [21]	China	n	kidney	29	f	n	n	n	48	ns	20	2	b	0	ns
Hara K et al., 2015 [22]	Japan	n	umbilical cord	30	f	n	n	n	1	MRI	100	2	c	0	ns
Lu J et al., 2016 [23]	China	n	bladder	46	m	n	n	n	1	CT	14	3	b	0	ns
John I et al., 2016 [23]	US	ns	paravertebral	85	m	n	n	n	1	ns	43	2	a	0	9
John I et al., 2016 [23]	US	ns	paravertebral	61	m	n	n	n	1	ns	ns	1		0	ns
John I et al., 2016 [23]	US	ns	paravertebral	67	m	n	n	n	1	ns	27	1		0	46
John I et al., 2016 [23]	US	ns	paravertebral	76	m	n	n	n	1	ns	15	1		0	32
John I et al., 2016 [23]	US	ns	paravertebral	31	f	n	n	n	1	ns	ns	3	a	0	ns
John I et al., 2016 [23]	US	ns	paravertebral	69	m	n	n	n	1	ns	42	1		0	ns
John I et al., 2016 [23]	US	ns	paravertebral	79	m	n	n	n	1	ns	75	2	a	0	12
John I et al., 2016 [23]	US	ns	paravertebral	67	f	n	n	n	1	ns	36	1		0	ns
John I et al., 2016 [23]	US	ns	paravertebral	67	m	n	n	n	1	ns	ns	1		0	12
John I et al., 2016 [23]	US	ns	retroperitoneal	67	m	n	n	n	1	ns	ns	1		0	12
John I et al., 2016 [23]	US	ns	mediastinum	70	f	n	n	n	1	ns	41	2	a	0	1
John I et al., 2016 [23]	US	ns	uterus	74	f	n	n	n	1	ns	67	2	a	0	ns
John I et al., 2016 [23]	US	ns	paravertebral	62	m	n	n	n	1	ns	24	2	a	0	1
John I et al., 2016 [23]	US	ns	uterus	36	f	n	n	n	1	ns	35	2	a	0	ns
John I et al., 2016 [23]	US	ns	paravertebral	53	m	n	n	n	1	ns	40	2	a	0	1
Dundr P et al., 2017 [24]	EU	y	ovary	66	f	n	n	n	1	ns	5	2	c	0	25
Dundr P et al., 2017 [24]	EU	y	ovary	43	f	n	n	n	1	ns	13	2	c	0	4
Dundr P et al., 2017 [24]	EU	y	ovary	69	f	n	n	n	1	ns	15	2	c	0	52
Dundr P et al., 2017 [24]	EU	y	ovary	81	f	n	n	n	1	ns	35	2	c	0	ns
Dundr P et al., 2017 [24]	EU	n	ovary	68	f	y	n	n	1	CT	35	2	c	0	ns
Dundr P et al., 2017 [24]	EU	y	ovary	69	f	y	n	n	1	ns	12	2	c	0	13
Burton KR et al., 2017 [25]	Canada	y	adrenal g.	68	m	y	y	n	120	CT	37	3	c	0	24
Sun k et al., 2017 [26]	China	y	liver	51	m	n	n	n	1	CT	50	2	b	0	36
Rodrigues MAS et al., 2017 [27]	Brazil	y	kidney	53	m	n	n	n	1	CT, MRI	25	2	a	0	ns
Abboudi H et al., 2017 [28]	EU	n	kidney	59	f	n	y	n	1	CT	27	2	c	0	36
Al Maghrabi HA et al., 2017 [4]	Saudi Arabia	n	kidney	55	f	n	y	y	1	CT	20	2	b	0	12
Perdiki M et al., 2017 [6]	EU	y	kidney	47	m	n	y	n	1	CT	25	2	c	0	14
Perdiki M et al., 2017 [6]	EU	n	kidney	64	f	n	n	n	1	CT	10	2	b	0	25
Berker NK et al., 2017 [29]	EU	y	kidney	24	f	n	y	n	1	MRI	30	2	b	0	10
Berker NK et al., 2017 [29]	EU	y	kidney	57	f	n	y	n	1	CT	26	2	c	0	4
Cheon PM et al., 2018 [30]	Canada	n	kidney	40	m	n	n	n	1	CT, MRI	46	2	c	0	12
Gunduz M et al., 2018 [31]	EU	y	ovary	62	f	n	n	n	1	CT	90	2	d	0	ns
Sagar R et al., 2019 [32]	US	y	kidney	39	f	n	y	y	1	CT	15	1		0	24
Lunn B et al., 2019 [33]	US	n	liver	33	f	n	n	n	1	CT, MRI	51	2	b	0	ns
Lunn B et al., 2019 [33]	US	n	liver	67	f	n	n	n	1	MRI	17	1		0	36
Lunn B et al., 2019 [33]	US	y	liver	77	m	n	n	n	1	CT, MRI	21	1		0	ns
Lunn B et al., 2019 [33]	US	y	liver	22	m	n	n	n	1	MRI	19	2	b	0	ns
Lunn B et al., 2019 [33]	US	y	liver	48	m	y	n	n	1	CT, MRI	17	3	b	0	ns
Merritt B et al., 2019 [34]	US	y	liver	56	m	y	n	n	1	CT, MRI	35	1		0	ns
Chandran N et al., 2019 [35]	India	y	kidney	36	m	n	y	n	1	CT	17	2	c	0	ns
Zheng LP et al., 2020 [36]	China	y	kidney	74	m	n	n	y	1	CT, MRI	26	2	b	0	2
Rathore K et al., 2020 [37]	Oceania	n	pericardium	64	m	n	n	y	1	CT, MRI	40	2	a	0	ns
Rezk A et al., 2020 [38]	US	y	ovary	60	f	n	n	n	12	CT	65	2	c	0	ns
Lin MS et al., 2020 [39]	US	y	breast	49	f	n	n	n	1	CT	10	2	a	0	12
Zhou J et al., 2020 [40]	China	n	kidney	28	m	n	n	n	1	CT	3,9	2	c	0	46
Zhou J et al., 2020 [40]	China	y	kidney	40	m	n	n	n	1	CT	0,8	2	c	0	10
Zhou J et al., 2020 [40]	China	y	kidney	45	f	n	n	n	1	CT	1,5	2	c	0	25
Zhou J et al., 2020 [40]	China	y	kidney	48	f	n	n	n	1	CT	1,8	2	c	0	30
Zhou J et al., 2020 [40]	China	y	kidney	52	m	n	n	n	1	CT	2	2	c	0	32
Zhou J et al., 2020 [40]	China	y	kidney	55	f	n	n	n	1	CT	2	2	c	0	33
Zhou J et al., 2020 [40]	China	y	kidney	60	m	n	n	n	1	CT	2,2	2	c	0	34
Zhou J et al., 2020 [40]	China	y	kidney	63	f	n	n	n	1	CT	2,5	2	c	0	38
Zhou J et al., 2020 [40]	China	y	adrenal g.	67	m	y	n	n	1	CT	2,8	2	c	0	40
Zhou J et al., 2020 [40]	China	y	adrenal g.	71	f	n	n	n	1	CT	30	2	c	0	42
Stewart CJR et al., 2020 [41]	Oceania	y	ovary	48	f	n	n	n	1	ns	8	2	c	0	ns
Johnstone KJ et al., 2020 [42]	Oceania	y	kidney	70	m	n	n	n	1	CT	35	2	c	0	ns
Kim et al., 2021 [43]	South Korea	y	kidney	35	m	n	y	y	1	CT, MRI	17	3	c	0	ns
Lo C et al., 2021 [44]	China	n	kidney	84	m	n	y	y	1	CT	55	2	c	2	1
Rogers T et al., 2022 [45]	US	y	liver	52	f	n	n	n	18	MRI	16	3	a	0	24
Shivani J et al., 2022 [46]	India	n	ovary	35	f	n	n	n	1	CT	110	2	c	0	ns
Chang Chien Y et al., 2022 [47]	EU	ns	ovary	68	f	n	n	n	1	ns	18	2	c	0	ns
Chang Chien Y et al., 2022 [47]	EU	ns	ovary	76	f	y	n	n	1	ns	35	2	c	0	ns
Chang Chien Y et al., 2022 [47]	EU	ns	kidney	52	f	n	n	n	1	ns	12	2	a	0	ns
Wei Ming C et al., 2022 [48]	Singapore	y	paravertebral	32	f	n	y	n	1	CT, RM, GaPET	18	1		0	ns
Nishikimi T et al., 2022 [49]	Japan	n	adrenal g.	49	m	n	n	n	4	CT	72	2	c	0	ns
Zhang Z et al., 2022 [50]	China	n	testis	84	m	n	n	n	1	CT, MRI	23	2	c	0	ns
Xue X et al., 2022 [51]	China	y	retroperit.	64	f	n	n	y	1	CT, MRI	108	2	a	0	24
Sasaki Y et al., 2023 [52]	Japan	y	kidney	65	m	n	y	n	36	CT, MRI	22	2	b	0	3
Ma Y et al., 2022 [53]	China	y	liver	29	m	n	n	n	72	CT, MRI	53	2	a	0	ns
Lazaro-Fontanet E et al., 2022 [54]	EU	y	liver	49	f	y	n	n	1	MRI	100	3	b	0	ns
Shaker N et al., 2022 [55]	US	n	retroperit.	66	m	n	n	n	1	CT	24	3	a	0	24
Wang Z et al., 2023 [56]	China	y	ovary	28	f	n	n	n	4	MRI	41	2	a	0	7
Ismayilov R et al., 2023 [57]	EU	y	liver	53	f	n	n	n	1	CT, MRI	25	2	a	0	6
Alaghehbandan R et al., 2023 [58]	US	y	adrenal g.	66	f	n	n	n	1	ns	8	2	c	0	5
Alaghehbandan R et al., 2023 [58]	US	y	adrenal g.	60	m	n	n	n	1	ns	15	2	c	0	140
Alaghehbandan R et al., 2023 [58]	US	n	adrenal g.	70	f	n	n	n	1	ns	17	2	c	0	12
Alaghehbandan R et al., 2023 [58]	US	n	adrenal g.	75	f	n	n	n	1	ns	7	2	c	0	156
Alaghehbandan R et al., 2023 [58]	US	y	adrenal g.	60	m	n	n	n	1	ns	64	2	c	0	95
Alaghehbandan R et al., 2023 [58]	US	y	adrenal g.	59	m	n	n	n	1	ns	10	2	c	0	ns
Alaghehbandan R et al., 2023 [58]	US	ns	adrenal g.	37	m	n	n	n	1	ns	27	2	c	0	ns
Yang L et al., 2023 [59]	China	n	liver	59	f	n	n	n	1	CT, MRI	100	2	a	0	12
Capinha MD et al., 2023 [60]	EU	y	kidney	70	m	n	y	y	3	CT, MRI	24	2	c	0	22
Slutsky HL et al., 2023 [61]	US	n	breast	37	f	n	n	n	1	US	10	3	a	0	36
Paparo AJ et al., 2023 [62]	Oceania	y	g.i. tract	55	m	n	n	n	1	ns	3	2	a	0	ns
McHenry A et al., 2023 [63]	US	n	ovary	55	f	n	n	n	1	CT	12	2	c	0	ns
McHenry A et al., 2023 [63]	US	n	ovary	62	f	n	n	n	1	CT	10	2	d	0	ns
McHenry A et al., 2023 [63]	US	y	ovary	67	f	y	n	n	1	CT	5	2	c	0	ns
McHenry A et al., 2023 [63]	US	y	ovary	76	f	y	n	n	1	CT	7	2	d	0	ns
McHenry A et al., 2023 [63]	US	y	ovary	58	f	n	n	n	1	CT	8	2	c	0	ns
McHenry A et al., 2023 [63]	US	n	ovary	53	f	y	n	n	1	CT	6	2	c	0	ns
McHenry A et al., 2023 [63]	US	y	ovary	73	f	y	n	n	1	CT	4	2	d	0	ns
McHenry A et al., 2023 [63]	US	y	ovary	65	f	n	n	n	1	CT	10	2	d	0	ns
McHenry A et al., 2023 [63]	US	y	ovary	50	f	y	n	n	1	CT	9	2	c	0	ns
McHenry A et al., 2023 [63]	US	y	ovary	69	f	y	n	n	1	CT	6	2	d	0	ns
McHenry A et al., 2023 [63]	US	y	ovary	63	f	n	n	n	1	CT	3	2	d	0	ns
McHenry A et al., 2023 [63]	US	y	ovary	55	f	y	n	n	1	CT	2	2	d	0	ns
Fang C et al., 2023 [64]	China	y	liver	50	f	n	n	n	94	CT, MRI	50	2	b	0	6
Zuo ZB et al., 2023 [65]	China	y	adrenal g.	46	m	n	n	y	1	CT	20	2	c	0	19
Caldwell NJ et al., 2024 [66]	US	n	mediastinum	59	f	n	n	n	1	CT, MRI, FDGPET	38	2	a	0	13
Caldwell NJ et al., 2024 [66]	US	y	mediastinum	55	f	n	n	n	29	CT, MRI, FDGPET	26	2	a	0	58
Caldwell NJ et al., 2024 [66]	US	n	mediastinum	72	f	n	n	n	1	CT, MRI, FDGPET	51	2	a	0	3
Caldwell NJ et al., 2024 [66]	US	ns	mediastinum	75	m	n	n	n	1	CT, MRI	ns	2	a	0	ns
Caldwell NJ et al., 2024 [66]	US	n	mediastinum	63	m	n	n	n	1	CT	56	2	a	0	ns
Caldwell NJ et al., 2024 [66]	US	n	mediastinum	77	f	n	n	n	11	CT	20	2	a	0	6
Caldwell NJ et al., 2024 [66]	US	n	mediastinum	72	f	n	n	n	1	CT	43	1		0	9
Wu Q et al., 2024 [67]	China	ns	ovary	26	f	n	n	n	5	CT	43	2	c	0	21
Present study	EU	y	retroperit.	68	m	n	n	y	1	CT, MRI, FDGPET	15	2	a	0	20

HTN: arterial hypertension; CT, computed tomography scan; EU, Europe; FDGPET, positron-emission tomography with fluorodeoxyglucose; MRI, magnetic resonance imaging; ns, not specified; US, United States. ^¥^ 1, observation after biopsy; 2, upfront surgery; 3, surgery after biopsy. * a, lesion resection; b, partial resection of the involved organ; c, total resection of the involved organ; d, extended resection.

## Data Availability

Data are available upon reasonable request.

## Abbreviations

The following abbreviations are used in this manuscript:

AH	Anastomosing Hemangioma
AS	Angiosarcoma
MRI	Magnetic Resonance Imaging
SUV	Standardized Uptake Value
